# Targeted deletion of keratin 8 in intestinal epithelial cells disrupts tissue integrity and predisposes to tumorigenesis in the colon

**DOI:** 10.1007/s00018-021-04081-5

**Published:** 2021-12-24

**Authors:** Carl-Gustaf A. Stenvall, Mina Tayyab, Tove J. Grönroos, Maria A. Ilomäki, Keijo Viiri, Karen M. Ridge, Lauri Polari, Diana M. Toivola

**Affiliations:** 1 Cell Biology, Biosciences, Faculty of Science and Engineering, Åbo Akademi University, BioCity, Tykistökatu 6A, N20520 Turku, Finland; 2 Turku PET Centre, University of Turku, Turku, Finland; 3 Medicity Research Laboratories, University of Turku, Turku, Finland; 4 Celiac Disease Research Center, Faculty of Medicine and Health Technology, Tampere University, Tampere University Hospital, Tampere, Finland; 5 Department of Cell and Developmental Biology, Feinberg School of Medicine, Northwestern University, Chicago, IL USA; 6 Turku Center for Disease Modeling, University of Turku, Turku, Finland

**Keywords:** Villin-Cre, Proliferation, Colon cancer, Tumorigenesis, Goblet cell, Notch, Barrier

## Abstract

Keratin 8 (K8) is the main intestinal epithelial intermediate filament protein with proposed roles for colonic epithelial cell integrity. Here, we used mice lacking K8 in intestinal epithelial cells (floxed K8 and Villin-Cre1000 and Villin-CreER^t2^) to investigate the cell-specific roles of intestinal epithelial K8 for colonocyte function and pathologies. Intestinal epithelial K8 deletion decreased K8 partner proteins, K18–K20, 75–95%, and the remaining keratin filaments were located at the colonocyte apical regions with type II K7, which decreased 30%. 2-Deoxy-2-[^18^F]-fluoroglucose positron emission tomography in vivo imaging identified a metabolic phenotype in the lower gut of the conditional K8 knockouts. These mice developed intestinal barrier leakiness, mild diarrhea, and epithelial damage, especially in the proximal colon. Mice exhibited shifted differentiation from enterocytes to goblet cells, displayed longer crypts and an increased number of Ki67 + transit-amplifying cells in the colon. Significant proproliferative and regenerative signaling occurred in the IL-22, STAT3, and pRb pathways, with minor effects on inflammatory parameters, which, however, increased in aging mice. Importantly, colonocyte K8 deletion induced a dramatically increased sensitivity to azoxymethane-induced tumorigenesis. In conclusion, intestinal epithelial K8 plays a significant role in colonocyte epithelial integrity maintenance, proliferation regulation and tumor suppression.

**Graphical abstract:**

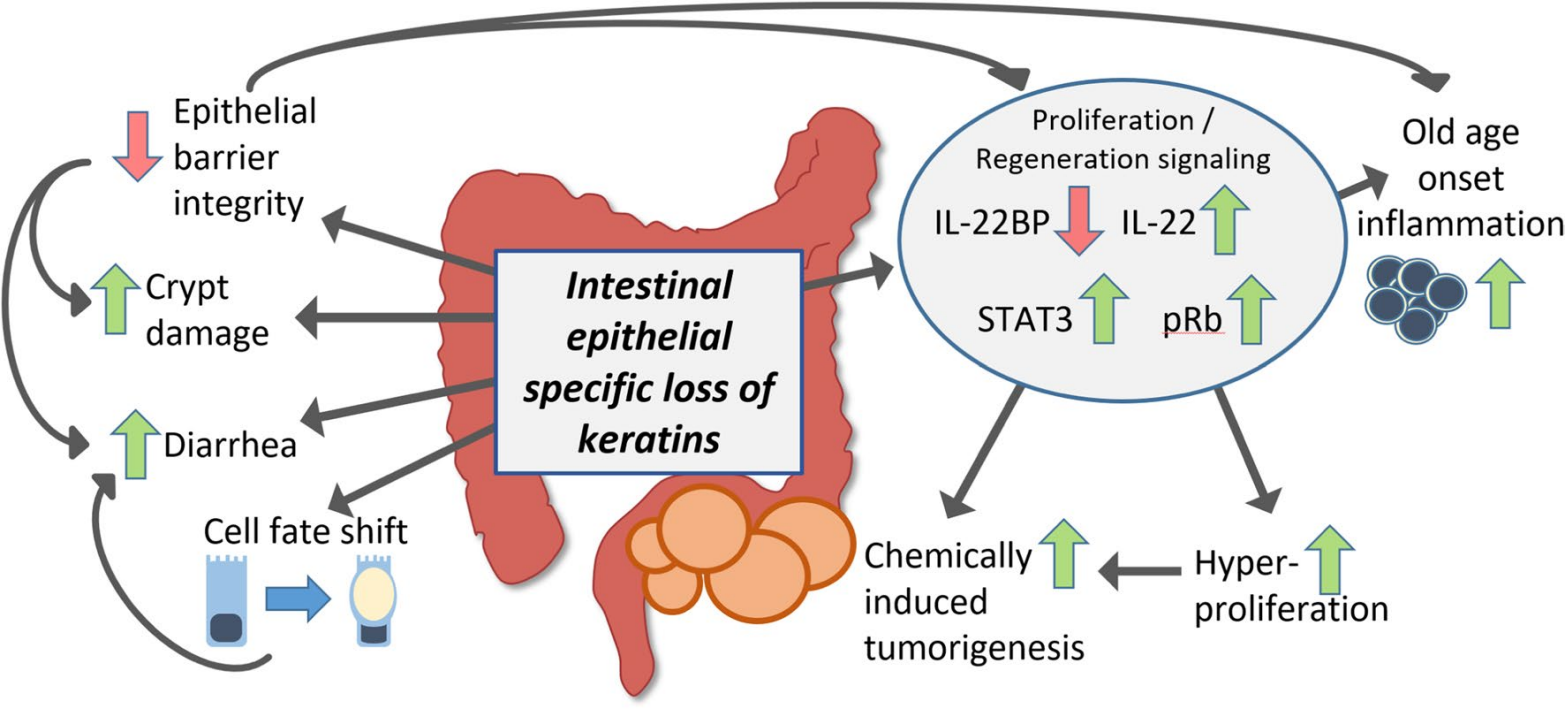

**Supplementary Information:**

The online version contains supplementary material available at 10.1007/s00018-021-04081-5.

## Introduction

The constant renewal of the epithelial cell layer in the intestinal mucosa ensures the maintenance of the barrier between the lumen and the submucosa. Loss of the barrier leads to a broad range of diseases, especially in the lower part of the gastrointestinal tract, including inflammatory bowel diseases (IBD) such as colitis, which can predispose to colorectal cancer (CRC) [1]. Keratins are mechanically strong intermediate filament (IF) proteins expressed in all epithelia and are formed by an assembly of obligate heteropolymers of type I and type II keratins. In simple type epithelia, such as the liver and intestine, the main members of the type I K18-K23 and type II K7-K8 simple epithelial keratin (SEK) family are expressed in a tissue type and differentiation-specific manner [2]. In both the small and large intestine, K8 is the major and probably the most important type II keratin [2], while K19 is its most abundant type I partner together with lower levels of K18 and K20 [3]. Additionally, in the mouse colon during basal conditions, minor levels of type II K7 is expressed [4], while in humans, K7 becomes expressed only in some human colorectal tumors [2]. In addition, SEK protein levels are dynamically regulated during intestinal stress conditions [5].

While IF and SEK variants are known to cause or predisposed to over 80 human diseases [6, 7], a correlation of SEK variants to intestinal disease has not been established even if a few cases have been described in IBD patients [8, 9]. As such, the colonocyte-specific roles of keratins in the multifactorial intestinal diseases are not well known. Several studies using K8-deficient mice where K8 is deleted in all K8 expressing cells (here called the K8^–/–^) [10, 11] support a role for keratins in the colon; however, the role for colonocyte K8 is not known. The whole body K8^–/–^ mice develop a colonic disease, manifested as an early-onset colitis phenotype with epithelial hyperproliferation, rectal prolapse, as well as defects in intestinal barrier, differentiation, metabolism and apoptosis [11–17]. In addition, K8^–/–^ are sensitive to chemically, as well as genetically, induced CRC [18].

Since K8 is the major type II keratin in all simple epithelia, the K8^–/–^ mouse also has multiple non-intestinal phenotypes, including for example high (50–95%) background strain-dependent embryo lethality, female sterility [10, 11], defective liver and β-cell function [19–21], and liver, gallbladder [22], placenta [23] and thyroid [24] deficiencies. In addition, aging K8^–/–^ male mice develop anti-mitochondrial serum autoantibodies [25], highlighting the systemic effects of the full body K8^–/–^. To investigate the colonocyte-specific roles of K8 in the intestinal epithelium and if the K8^–/–^ colonic phenotype is caused by colonocyte-specific keratin dysfunction, we have here used the cre-loxP system (using Villin-Cre mice [26]) to generate a mouse model deficient in K8 in villin-expressing intestinal epithelial cells. We show here that this conditional intestinal epithelium-specific K8 deletion induces colonic hyperproliferation, crypt damage, diarrhea, leaky epithelial barrier, and high sensitivity to azoxymethane (AOM)-induced tumorigenesis.

## Results

### Intestinal epithelial-specific K8 deletion is accompanied by downregulation of the other main colonic epithelial keratins

**Fig. 1:**
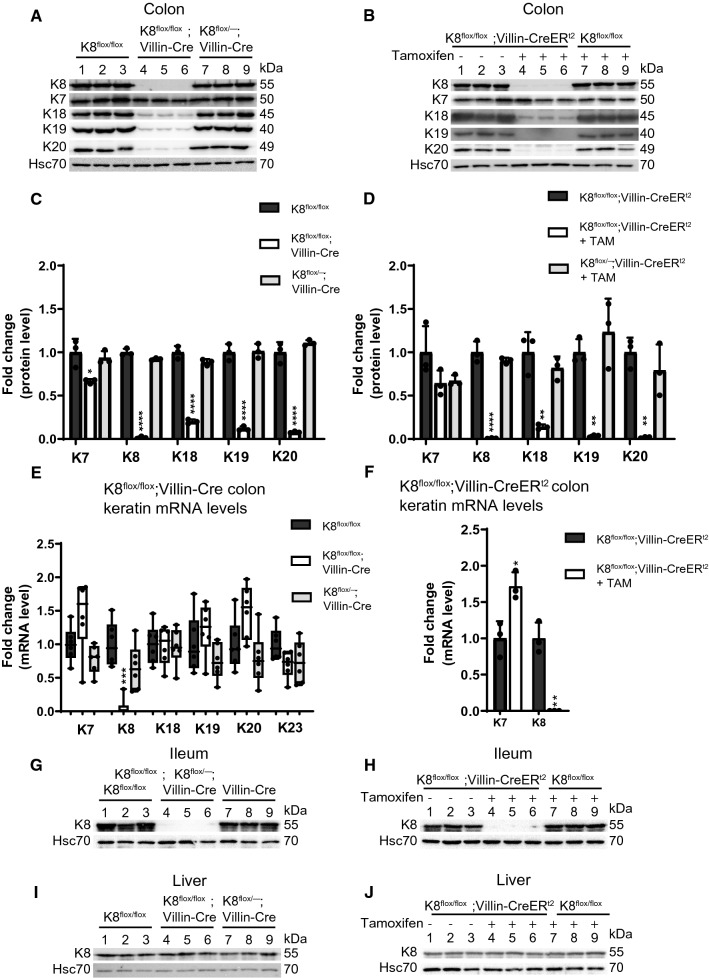
Intestinal epithelial-specific K8 deletion induces local keratin loss in intestinal epithelia.** A** Lysates of crudely isolated colon epithelium from K8^flox/flox^ (lane 1–3), K8^flox/flox^; Villin-Cre (lane 4–6) and K8^flox/–^; Villin-Cre (lane 7–9) mice (*n* = 3) were immunoblotted for K8, K7, K18, K19 and K20. **B** Total colon lysates from untreated K8^flox/flox^; Villin-CreER^t2^ (lane 1–3), and tamoxifen-treated (25 days after first injection) K8^flox/flox^; Villin-CreER^t2^ (lane 4–6) and K8^flox/flox^ (lane 7–8) mice (*n* = 3) were immunoblotted for K8, K7, K18, K19 and K20. Hsc70 was used as a loading control for both **A** and **B**. **C**, **D** The immunoblots in A and B were quantified and normalized to Hsc70. The results represent the mean (*n* = 3) protein quantity ± SD with significant differences shown between K8^flox/flox^ and K8^flox/flox^; Villin-Cre mice (**C**), and between untreated and tamoxifen-treated K8^flox/flox^; Villin-CreER^t2^ mice (**D**), with individual values shown as dots. **E**, **F** The mRNA levels of *Krt8*, *Krt7* (**E** and **F**) and *Krt18*, *Krt19*, *Krt20* and *Krt23* (**E**) of intestine-specific K8 knockout mice total colon lysates were analyzed by qRT-PCR. The results were normalized to both *Actb* and 18S ribosomal RNA expression and boxes (**E**) extend from 25 to 75th percentiles and line represents median expression value and whiskers represent min and max values with individual mice values shown as dots (*n* = 6), while (**F**) shows the average (*n* = 3) fold change ± SD and individual values shown as dots. **G**, **H** Ileum and **I**, **J** liver total lysates from intestine-specific K8 knockout mice were immunoblotted for K8 and Hsc70 was used as a loading control. The statistical significance was determined after one-way ANOVA, followed by post hoc Tukey multiple comparison test, expect in **F** by student’s *T* test, and shown as *P < 0.05, **P < 0.01, ***P < 0.001 and ****P < 0.0001

To investigate the cell-specific role of K8 in colonic epithelial cells, we established a tissue-specific conditional K8 knockout mouse model using the loxP-Cre recombinase system (Supplemental Fig. 1). By utilizing Villin-Cre transgenic mice, K8 should be lost in crypts and villi of the small and large intestine of K8^flox/flox^ mice. For this purpose, we generated two models. K8^flox/flox^; Villin-Cre1000 (here called K8^flox/flox^; Villin-Cre) are mice with the K8 deletion starting in the embryo, and K8^flox/flox^; Villin-CreER^t2^ mice requiring tamoxifen administrations (25 days after tamoxifen administration) to induce K8 deletion [26]. Both conditional K8 knockout models showed complete loss of K8 (Fig. 1A, B) when crudely isolated colonic epithelial tissue lysates (Fig. 1A) or total colon tissue (Fig. 1B) were analyzed by western blotting. Additionally, a significant downregulation of all main colonic type I keratins K18, K19 and K20 was found (Fig. 1A–D). Only K8, but not K18, K19 or K20 mRNA, levels were decreased in both K8 conditional knockout mouse lines (Fig. 1E, F), showing that the K8 deletion does not affect transcription of K8 partners. Interestingly, K7 protein levels were only marginally reduced (~ 30%) in the K8^flox/flox^; Villin-Cre colon and slightly upregulated on mRNA level in K8^flox/flox^; Villin-CreER^t2^ (Fig. 1A, E–F). K8 was also deleted in the ileum, but not in liver (which displayed no histological abnormalities), as expected (Fig. 1G–J; Supplemental Fig. 2), confirming that the deletion of K8 is faithful to villin-expressing cells. Furthermore, the histology of K8^flox/flox^; Villin-Cre kidney tubules, uterus and gallbladder epithelia, which express minor levels of villin, appeared normal (Supplemental Fig. 2) [26, 27]. K8^flox/flox^; Villin-Cre female mice were fertile, and no embryo lethality was noticed (Supplemental Table 1) (when K8^flox/flox^; Villin-Cre and K8^flox/flox^ were bred, 50% offspring were genotyped as K8^flox/flox^; Villin-Cre). Keratin immunostainings confirmed the complete loss of K8 in the K8^flox/flox^; Villin-Cre colonic epithelium (Supplemental Fig. 3A), and a significant decrease of K7, K18 and K19 throughout the crypt with remaining staining in the apical compartment of colonic epithelial cells (Supplemental Fig. 3A, B).

### Intestine-specific K8 knockdown leads to increased intestinal permeability, diarrhea, colonic epithelial damage and crypt length increase

**Fig. 2:**
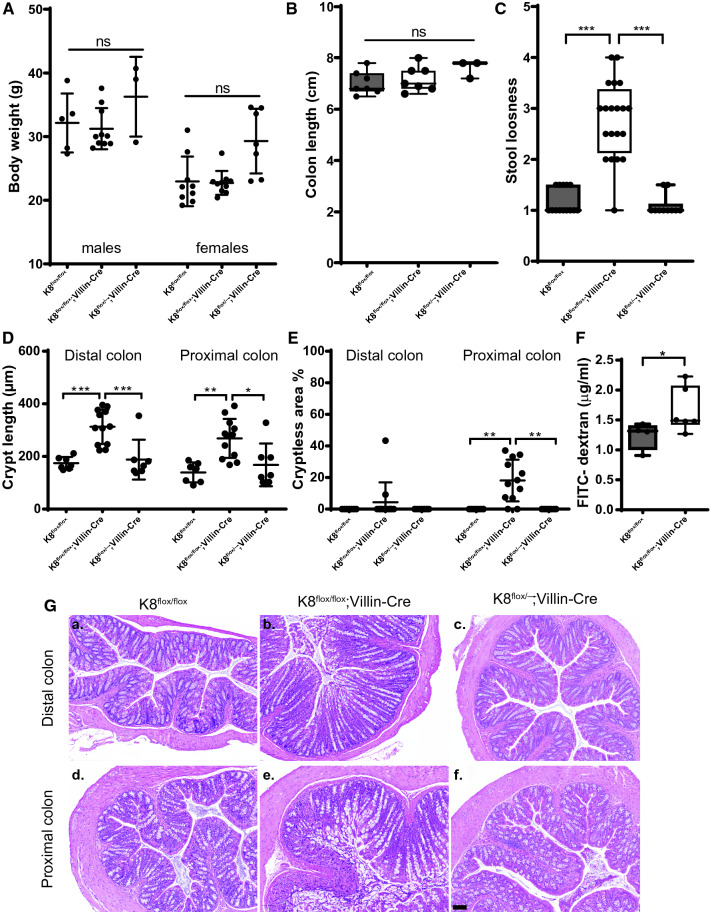
Intestinal epithelial specific K8 knockout mice develop colonic epithelial damage, loss of barrier and diarrhea. K8^flox/flox^, K8^flox/flox^; Villin-Cre and K8^flox/–^; Villin-Cre mice were analyzed for body weight (**A**), colon length (**B**), stool looseness (**C**), distal and proximal colon crypt length (**D**), distal and proximal cryptless areas (**E**), and colon barrier permeability (for K8^flox/flox^ and K8^flox/flox^; Villin-Cre mice) (**F**). *n* = 9–20 mice 3–8 months of age and dots indicate individual mice and boxes in **B**, **C**, **F** extend from 25 to 75th percentiles and line represents median value and whiskers represent min and max values. **G** Representative HE stainings of distal and proximal colon from K8^flox/flox^, K8^flox/flox^; Villin-Cre and K8^flox/–^; Villin-Cre mice. Scale bar 100 µm. *P* values represent difference between genotypes and were determined after one-way ANOVA followed by post hoc Tukey multiple comparison test, except **F**, which was determined by Kolmogorov–Smirnov test. *n*.*s*. = not significant, *P < 0.05, **P < 0.01 and ***P < 0.001

While the K8^flox/flox^; Villin-Cre or K8^flox/–^; Villin-Cre young adult mice did not differ in body weight or in colon length compared to K8^flox/flox^ control mice (Fig. 2A, B), the K8^flox/flox^; Villin-Cre stool consistency was significantly softer compared to both K8^flox/flox^ and K8^flox/–^; Villin-Cre mice (Fig. 2C) indicating mild diarrhea. Histological analysis showed on average a 1.5–2-fold increased crypt length in the proximal and distal colon of K8^flox/flox^; Villin-Cre mice, while deletion of one allele in K8^flox/–^; Villin-Cre did not affect K8 protein levels (Fig. 1C, D), crypt length or stool consistency (Fig. 2C, D, G).

Wide colonic epithelial damage was observed in the K8^flox/flox^; Villin-Cre mice, predominately in the proximal colon where the damage measured as % cryptless areas represented on average 20%, and up to 40% of total epithelium (Fig. 2E, G). The K8^flox/–^; Villin-Cre mice displayed no crypt loss (Fig. 2E, G). Similarly, K8 knockdown in colons from adult K8^flox/flox^; Villin-CreER^t2^ mice 25 days after first tamoxifen injection induced a milky appearance of the colon without distinguishable stool pellets (Supplementary Fig. 4A) and similar increase in colon crypt lengths and epithelial erosion (Supplementary Fig. 4B–D) as in the K8^flox/flox^; Villin-Cre mice. Tamoxifen treatment did not induce crypt length changes or colon epithelia loss in control K8^flox/flox^ and Villin-CreER^t2^ mice (Supplementary Fig. 4B–D). Comparison of distal and proximal colon phenotypes in conditional and K8^–/–^ knockout models in a subset of age-matched mice showed similar increases in crypt length across the models and colon segments, with the longest average crypt lengths in the K8^–/–^ (Supplemental Fig. 4 E–F). The crypt loss phenotype was seen in all models, but was more pronounced in the conditional K8 knockout models (Supplemental Fig. 4G–H). K8-deficiency induced crypt damage suggested a disruption of the colonic barrier function and, indeed, fluorescein isothiocyanate-conjugated dextran (FITC–dextran FD4) in vivo permeability assay measured 6 h after oral gavage revealed an increased permeability in K8^flox/flox^; Villin-Cre mice compared to K8^flox/flox^ mice (Fig. 2F).

### Intestinal epithelial-specific K8 deficiency promotes age-dependent inflammatory responses

Colitis is commonly associated with leukocyte infiltration and, thus, increased inflammatory responses [28]. Since K8^flox/flox^; Villin-Cre mice develop mild diarrhea, colon epithelial damage and barrier brake similar to those in colitis, we next characterized the levels of inflammatory mediators and immune cells in these mice. No major systemic inflammation was observed in 3- to 8-month-old K8^flox/flox^; Villin-Cre mice, as circulating inflammatory mediators chemokine ligand 2 (CCL-2), TNF, interferon (IFN)γ, IL-6, IL-5, and IL-18 were close to basal levels, with only IL-1β showing a statistically significant, but still modest, increase compared to K8^flox/flox^ (Supplemental Fig. 5A). In addition, IL-22 serum levels were on average slightly higher in K8^flox/flox^; Villin-Cre compared to K8^flox/flox^, but did not reach statistical significance (*P* = 0.099), while IL-25 was below the detection limit (3.8 pg/ml). In comparison, K8^–/–^ mice showed increased levels of IL-1β, TNFα, IL-6, IL-5, IL-22, and lower levels of IL-18 in blood serum compared to K8^+/+^ mice (Supplemental Fig. 5D). No clear increase in local colon inflammation of K8^flox/flox^; Villin-Cre mice could be found either, as the mRNA levels of macrophage and T cell-produced substances such as CCL-2, IL-1β, IL-4 and IL-6 were unaffected in total colon lysates (Supplemental Fig. 5B). IL-18 mRNA synthesis in the K8^flox/flox^; Villin-Cre colon was slightly reduced, suggesting that there was no major increase in the number or activity of T cells and antigen presenting cells (Supplemental Fig. 5B). This finding is supported by histological analysis showing no major flux of monocytes or neutrophils inside the muscularis mucosae (Fig. 2G). Despite slightly higher average levels, there was no statistically significant increase in myeloperoxidase (MPO) mRNA levels (Supplemental Fig. 5B) or MPO+ cells (Supplemental Fig. 5C), supporting that colon neutrophil number stay close to basal level in K8^flox/flox^; Villin-Cre mice.

**Fig. 3:**
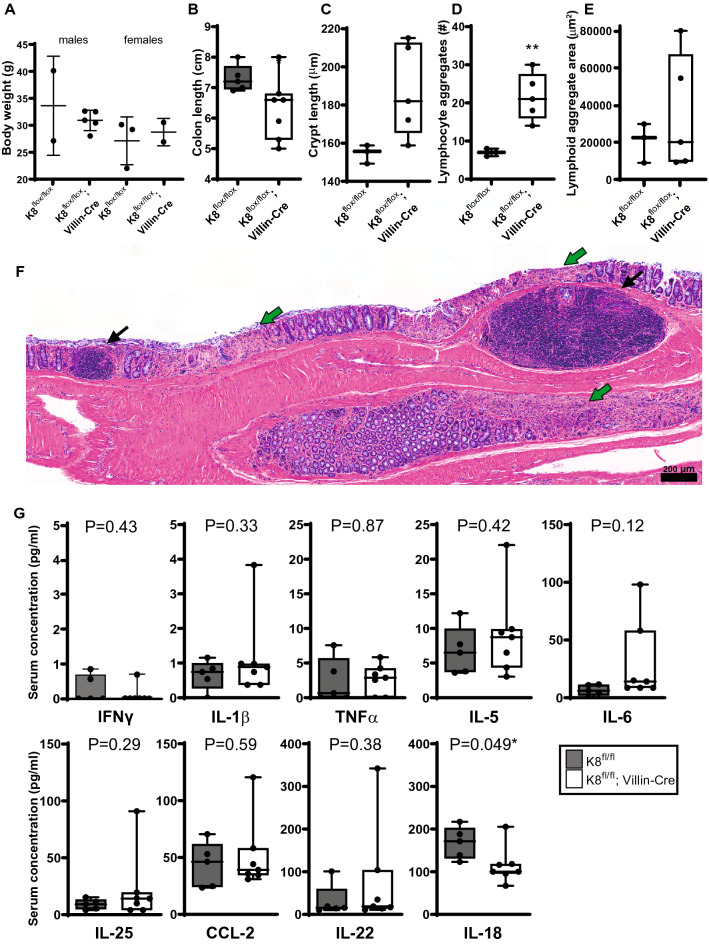
The K8 loss induced inflammation phenotype is more pronounced in mice over 250 days old. K8^flox/flox^ and K8^flox/flox^; Villin-Cre male and female mice over 250 days old were analyzed for body weight, dots representing a single mouse (**A**), colon length (**B**), average crypt length (**C**), the number of lymphoid cell aggregates in longitudinal colon cuts (**D**) and the mean area of lymphoid aggregates (**E**). **F** A representative colon section of an older K8^flox/flox^; Villin-Cre mouse in which black arrows show lymphoid aggregates and green arrows epithelial erosion. Scale bar 200 µm. **G** The circulating concentrations of serum cytokines IFNχ, IL-1β, TNFα, IL-5, IL-6, IL-25, CCL-2 IL-22, and IL-18 of > 250 days old mice measured using Luminex immunoassay. Boxes extend from the 25 to 75th percentiles and the line represents the median expression value, whiskers represent min and max values and individual mice values are represented as dots. *P* values and asterisks (*P < 0.05 and **P < 0.01) represent statistical difference, calculated using Student’s *t* test. *n* = 3–5

However, in 10- to 15-month-old K8^flox/flox^; Villin-Cre mice, a colonic inflammatory phenotype became more predominant, when compared to age-matched K8^flox/flox^ mice of roughly the same body weight (Fig. 3A). This was seen as a ~ 15% shortened colon as well as a threefold increased number of lymphatic cell aggregates in the colon (Fig. 3B, D, E). Interestingly, the older K8^flox/flox^; Villin-Cre mice exhibited only a minor crypt length increase compared to age-matched K8^flox/flox^ (Fig. 3C), but still showed crypt and epithelial erosion (Fig. 3F). Both control K8^flox/flox^ and K8^flox/flox^; Villin-Cre aged mice showed an upregulation of some circulating cytokines, especially IL-22, IL-25 and TNFα (Fig. 3G) compared to the 3-to 8-month-old mice (see Supplemental Fig. 5A); however, no differences between genotypes were seen due to a high individual variation in the aged K8^flox/flox^; Villin-Cre mice, apart from IL-18, which was lower in 10- to 15-month-old K8^flox/flox^; Villin-Cre compared to controls (Fig. 3G).

### 2-Deoxy-2-[^18^F]fluoroglucose position emission tomography imaging detects increased metabolic activity in the lower gut of K8^flox/flox^; Villin-Cre mice

**Fig. 4:**
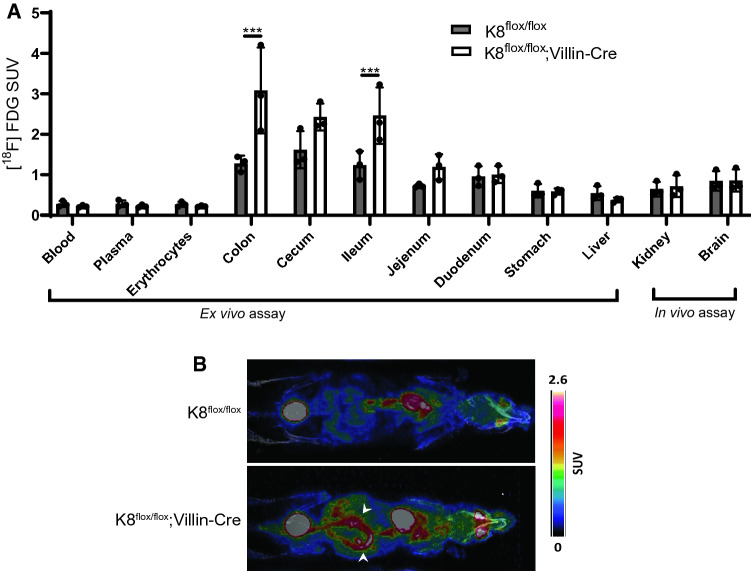
K8^flox/flox^; Villin-Cre mice possess increased metabolic activity in the lower gut, measured using [^18^F]FDG-PET in vivo and ex vivo imaging. K8^flox/flox^ and K8^flox/flox^; Villin-Cre mice (7–8 months old) were injected with [^18^F]FDG in the tail vein and imaged after 1 h. **A** ^18^F-radioactivity was measured in blood, plasma, erythrocytes, colon, ceacum, ileum, jejunum, duodenum, stomach, and liver ex vivo and in kidney and brain from in vivo images. Significant radioactivity was observed in the colon and ileum of K8^flox/flox^; Villin-Cre mice compared to K8^flox/flox^ mice. Bars represent mean SUV ± SD and individual mice values are shown as dots. **B** Representative images indicate increased metabolic activity in the lower gut, where arrows highlight the increased activity in colon tissue. SUV is standard uptake value. *P* values represent the difference between genotypes and were determined using Student’s *t* test. ***P < 0.001. *n* = 3

To analyze the metabolic activity, which increases in colon during inflammatory conditions, K8^flox/flox^; Villin-Cre and K8^flox/flox^mice were injected with 2-deoxy-2-[^18^F]fluoroglucose ([^18^F]FDG) in the tail vein. In vivo positron emission tomography (PET) analysis showed an [^18^F]FDG accumulation in the K8^flox/flox^; Villin-Cre compared to K8^flox/flox^ colon (Fig. 4A, B). The ^18^F-radioactivity measurements in different organs collected ex vivo after PET imaging confirmed the threefold increase in radioactivity in the K8^flox/flox^; Villin-Cre colon compared to K8^flox/flox^, with slightly lower uptake increase in the ileum. Other studied organs had comparable uptake in both genotypes (Fig. 4B).

### Loss of intestinal K8 leads to a shifted differentiation and increased proliferation in colonocytes

**Fig. 5:**
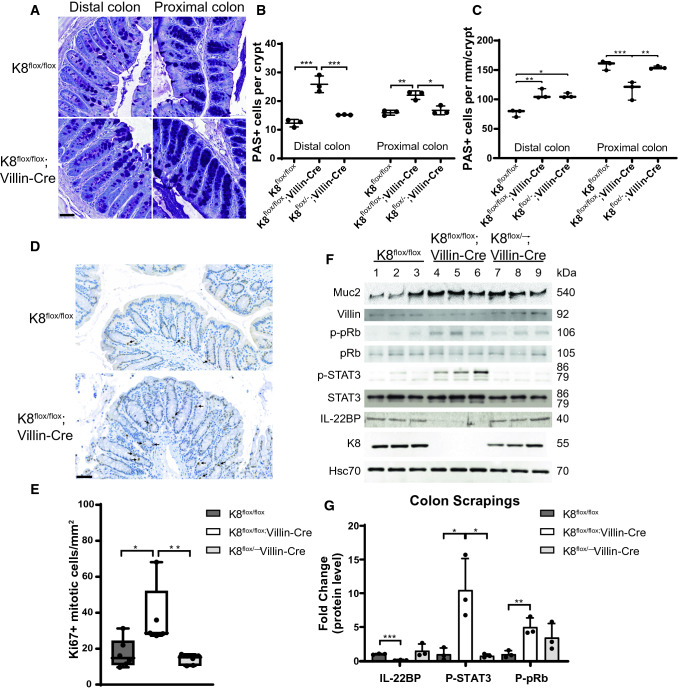
Local keratin deficiency in the colon is accompanied by increased cell proliferation and increase in goblet cells. **A** Representative distal and proximal K8^flox/flox^ and K8^flox/flox^; Villin-Cre colon images stained with PAS. Scale bar = 50 µm. **B** Number of goblet cells per crypt and **C** per millimeter of crypt in distal and proximal colon from PAS-stained K8^flox/flox^, K8^flox/flox^; Villin-Cre and K8^flox/–^; Villin-Cre mice (*n* = 3). **D**, **E** Representative Ki67-stained K8^flox/flox^ and K8^flox/flox^; Villin-Cre colon images and the difference of Ki67-positive mitotic bodies per mm^2^ between genotypes (*n* = 6). Arrows indicate mitotic bodies in C; boxes extend from 25 to 75th percentiles and line represents median and whiskers min and max values in **D**. Scale bar = µm. **F** Lysates of crudely isolated colon epithelium from K8^flox/flox^ (lane 1–3), K8^flox/flox^; Villin-Cre (lane 4–6) and K8^flox/–^; Villin-Cre (lane 7–9) mice (*n* = 3) were immunoblotted for Muc2, Villin, p-pRb, pRb, p-STAT3, STAT3, IL22BP and K8. Hsc70 was used as a loading control. **G** IL-22BP, p-STAT3 and p-pRb immunoblots were quantified and normalized to Hsc70 (IL-22BP), STAT3 (p-STAT3) or pRb (p-pRb). Boxes extend from the 25th to 75th percentiles and the line represents median expression value, whiskers represent min and max values and individual mice values are represented as dots. *P* values represent the difference between genotypes and were determined after one-way ANOVA, followed by post hoc Tukey multiple comparison test. *P < 0.05, **P < 0.01 and ***P < 0.001

Next, we analyzed whether the intestine-specific loss of K8 affects the colonocyte differentiation and proliferation levels. Quantification of colon goblet cells according to the periodic acid Schiff (PAS) staining (Fig. 5A–C) of adult mice showed that the number of PAS-positive cells per crypt was increased in K8^flox/flox^; Villin-Cre colon. When the number of goblet cells was normalized to crypt length, their density per millimeter was significantly higher in the distal colon while they were decreased in the proximal colon (Fig. 5C). An increase in the goblet cell protein mucin 2 (Muc2) supported an overall higher number of goblet cells in K8^flox/flox^; Villin-Cre mice (Fig. 5F). Villin protein levels were decreased in K8^flox/flox^; Villin-Cre mice, indicating a decrease in enterocytes (Fig. 5F) and a colonocyte K8-dependent shift in cell fate. K8 has been shown to interact with Notch1 and affect its activity, thereby shift colonic differentiation from enterocytes to goblet cells^17^. We next analyzed full-length Notch1 (FLN) and Notch1 intracellular domain (NICD) protein levels in the conditional K8 knockout models. FLN, but not NICD, protein levels were decreased in the tamoxifen-treated K8^flox/flox^; Villin-CreER^t2^ mice (Supplemental Fig. 6C, D). Furthermore, FLN immunostaining in K8^flox/flox^ and K8^flox/flox^; Villin-Cre proximal and distal colon showed decreased fluorescence intensity in K8^flox/flox^; Villin-Cre mice (Supplemental Fig. 6E).

To analyze if the K8^flox/flox^; Villin-Cre increased crypt length was due to hyperproliferation, we analyzed the number of Ki67+ mitotic bodies inside colon crypts (Fig. 5D, E). The number of dividing Ki67+ cells were dramatically increased in K8^flox/flox^; Villin-Cre mouse epithelium, but not in K8^flox/−^; Villin-Cre (Fig. 5D, E), indicating that loss of both colonocyte K8 alleles is needed in this model to induce epithelial cell hyperproliferation. To study the signaling behind the K8-dependent epithelial proliferation, the activity of the STAT3 pathway was assessed by analyzing the phosphorylation of STAT3 (tyrosine 705), as well the levels of the upstream IL-22BP protein (an IL-22 binding protein that limits IL-22 signaling), which further increases STAT3 activation in intestinal epithelia [29], thus increasing proliferation. Colonic IL-22BP levels were strongly decreased, and p-STAT3 levels (Fig. 5F, G) and its STAT3 target gene S100A11 (Supplementary Fig. 6A) were increased in K8^flox/flox^; Villin-Cre epithelium, but not in K8^flox/−^; Villin-Cre, compared to K8^flox/flox^ controls. Similar IL-22BP loss and STAT3 activation were seen 25 days after onset of K8 deletion in tamoxifen-treated K8^flox/flox^; Villin-CreER^t2^ mice (Supplementary Fig. 6C, D). We next analyzed the phosphorylation state of the nuclear retinoblastoma protein (pRb) which is a central negative regulator of the cell cycle, where its phosphorylation at serines 807/811 inhibits pRb activity and thereby promotes cell cycle progression. Indeed, p-pRb levels were increased in the K8^flox/flox^; Villin-Cre colon, and while K8^flox/−^; Villin-Cre mice had on average higher levels, they were not significantly altered (Fig. 5F, G). The activation of the pRb pathways was seen by an increased mRNA level of the pRb target gene *Mybl2* in K8^flox/flox^; Villin-Cre mice colon tissue (Supplemental Fig. 6B)*.* Taken together, K8 deletion from intestinal epithelial cells stimulates proliferation pathways in the colon.

### Local keratin dysregulation sensitizes to colon carcinogenesis

**Fig. 6:**
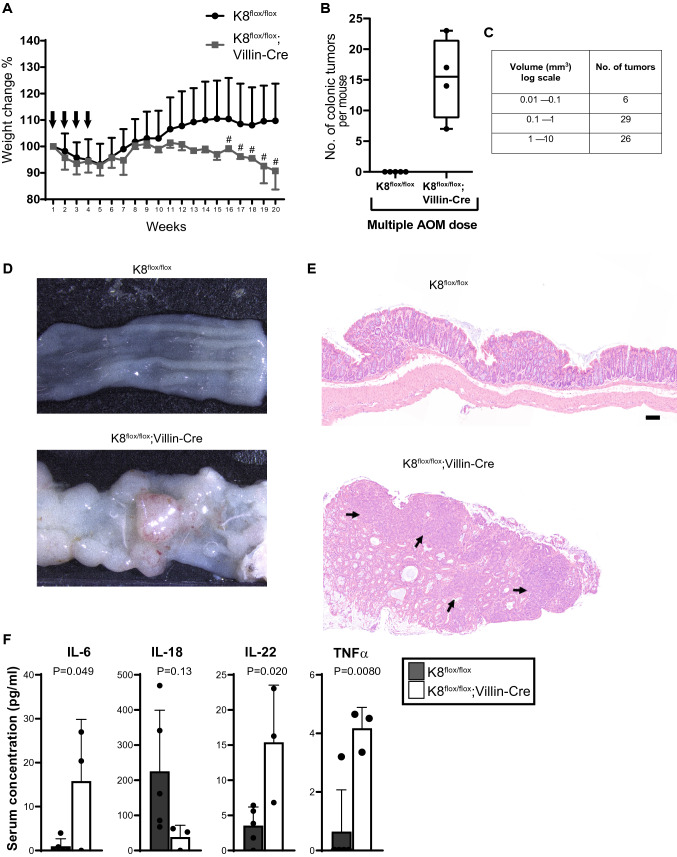
Intestine-specific K8 deletion sensitizes mice to chemically induced tumorigenesis in the distal colon. K8^flox/flox^ and K8^flox/flox^; Villin-Cre mice were intraperitoneally injected with 10 mg/kg of AOM once per week for 4 weeks (arrows in **A**) and then killed after 20 weeks. **A** K8^flox/flox^ and K8^flox/flox^; Villin-Cre body weight changes during AOM treatment are shown as average ± SD and # indicates onset and occurrence of soft stool and blood in the stool of K8^flox/flox^; Villin-Cre mice. **B** The average number of colonic tumors per genotype from four mice per genotype where boxes extend from the 25th to 75th percentiles, line represents median expression value and whiskers represent min and max values with individual values represented as dots. **C** The number of K8^flox/flox^; Villin-Cre mouse tumors and size (volume calculated and shown as log scale mm^3^). **D** Representative images of distal colon after AOM treatment from K8^flox/flox^ and K8^flox/flox^; Villin-Cre are shown. **E** HE-stained colon images show the epithelial origin of tumors, where arrows represent the tumor areas. Scale bar 100 μm. **F** Serum concentrations for IL-6, IL-18, IL-22 and TNFα were measured in K8^flox/flox^ and K8^flox/flox^; Villin-Cre mice. Error bars represent SD with individual mice represented as dots. *P* values and asterisks (*P < 0.05 and **P < 0.01) represent statistical difference calculated using Student’s *t* test. *n* = 4–5

Since K8^flox/flox^; Villin-Cre mice exhibited a robust increase in colonic crypt length and colonocyte proliferation, while an increase in inflammatory mediators was close to negligible in younger adult mice, we assessed whether these changes are still enough to affect susceptibility to colorectal tumor development. No intestinal tumors were observed macroscopically or histologically in untreated K8^flox/flox^; Villin-Cre mice, although these mice developed occasional prolapse of the rectum similar to the K8^–/–^ mice (Supplemental Table 1). However, AOM administration (10 mg/kg AOM to 5-month-old mice, once per week for 4 weeks, Fig. 6A) strongly promoted carcinogenesis in K8^flox/flox^; Villin-Cre mice (Fig. 6), with an average of 15 tumors in the distal colon after 20 weeks of the initial AOM administration (Fig. 6B), while K8^flox/flox^ mice did not develop any visible tumors. Most K8^flox/flox^; Villin-Cre tumor volumes ranged from 0.1 mm^3^ to over 10 mm^3^ (Fig. 6C), and histological analysis revealed the epithelial origin of the tumors (Fig. 6E). The tumor development in AOM-treated K8^flox/flox^; Villin-Cre mice, but not K8^flox/flox^ mice correlated with body weight loss starting from week 13 and rectal bleeding after week 16 (Fig. 6A). The AOM-treated K8^flox/flox^; Villin-Cre mice had also notable changes in the circulating cytokine levels, as IL-6, IL-22 and TNFα were higher compared to AOM-treated K8^flox/flox^ mice (Fig. 6F).

## Discussion

In this study, we demonstrate the importance of the main colonocyte intermediate filament K8 for colon health in vivo*,* utilizing mouse models where the main colonic epithelial type II keratin K8 was deleted from intestinal epithelial cells. K8^flox/flox^; Villin-Cre mice and K8^flox/flox^; Villin-CreER^t2^ mice after 25 days of tamoxifen induction in adult mice expressed no intestinal epithelial K8, and the other main K8 type I partners K18, K19 and K20 decreased nearly completely, similarly to what has been shown in the K8^–/–^ mouse [10, 11, 15]. Type II K7 protein levels decreased more modestly in the conditional K8-knockout colon compared to the full K8^–/–^ mice. In contrast to previous findings where K7 was found restricted to the mid and lower part of the mouse crypts [4, 30], the present study shows that K7 is expressed throughout the crypt in the normal mouse colon. In the K8^flox/flox^; Villin-Cre mice, the remaining K7 localizes with K18 and K19 at the apical cell membrane, indicating that the reduced type II K7 supports the residual presence of type I keratins when K8 is missing. These residual keratins are likely essential to maintain the most necessary cellular integrity for survival at the apical membrane where colonic keratins are prominent [31].

Using the keratin-deficient intestinal epithelial specific K8 knockout model developed here, we report that loss of K8 only in these cells leads to major colonic disease phenotypes including: (i) partial loss of colonic epithelium, (ii) compromised intestinal barrier and diarrhea, (iii) increased metabolic activity and (iv) a modest colonic inflammation, which is more pronounced in aging mice. On cellular level, the intestine-specific K8 deletion leads to (v) a shifted colonocyte cell differentiation toward a goblet cell fate linked to decreased Notch1; (vi) an increased proliferation and regeneration capacity seen as longer crypts, occasional prolapse, and increased cell proliferation signaling, as well as (vii) a dramatic increase in susceptibility to chemically induced colorectal cancer. Since K8 is also expressed, e.g., in the liver and uterus in mice [3], In addition, female K8^flox/flox^; Villin-Cre mice are fertile and no embryo lethality was observed in contrast to K8^–/–^ mice [10, 11]. These findings confirm that the colonic K8^flox/flox^; Villin-Cre phenotypes are solely induced by intestinal epithelial cell keratins. Since the colonic hyperproliferation [11], cell fate switch [17] and tumorigenesis susceptibility[18] described in the full K8^–/–^ closely resembling that of the intestinal epithelial-specific K8 mice described here (comparisons on colon disease and molecular phenotypes are listed in Supplemental Tables 1 and 2), the current study strongly underlines the importance of colonic epithelial keratins for colon health and homeostasis.

One notable difference between the K8^flox/flox^; Villin-Cre and the K8^–/–^ mouse models was the immune cell activity. K8^flox/flox^; Villin-Cre had only a minor systemic or local immunological responses, witnessed by slight increase in circulating IL-1β and IL-22 and the number of MPO expressing neutrophils in the colon was not changed. Despite the significant epithelial damage, the changes in immune cell number and gene expression in colon mucosa remained surprisingly low, while the K8^–/–^ mouse displayed lymphocyte and neutrophil infiltration [13, 15]. In contrast, older K8^flox/flox^; Villin-Cre mice had a more pronounced colitis phenotype as seen by shortened colon and increased number of lymphocyte aggregates in colon compared to age-matched controls and young adult K8^flox/flox^; Villin-Cre mice. Still, these changes did not induce an active Th2 type inflammation [13] as suggested by unaltered circulating IL-5 and IL-25 concentrations. K8^flox/flox^; Villin-Cre lived at least to 15 months of age without showing any additional signs of premature infirmity.

Aging itself can also modulate epithelial keratin levels [5], but the effect on immune cells is not well studied. The age-induced increase in some of the circulating cytokines such as TNFα and IL-25 can be associated with age-induced shifts in both subset changes in macrophages and T cells as well as in colon microbiota [32–34]. Importantly, it can be assumed that the more robust and early inflammatory response in the K8^–/–^ mouse colon[13] is at least partially reflected by other keratin-deficient simple epithelial organ failures such as the major tissue fragility described in the liver [35, 36]. The K8^–/–^ mouse develops not only a colitis-like phenotype, but also changes, e.g., in glucose metabolism, insulin secretion and liver fragility, thereby obscuring what causes the colon phenotype [19, 20]. We also cannot exclude the role of the different background mouse strains in the K8^–/–^ (FVB/n) and the intestine-specific K8-knockout models (C57Bl6), as immunological difference are known to exist between the strains [37], and is a limitation of the study comparing the inflammatory phenotype to the K8-full knockout model. K8^–/–^ mouse has been previously listed as a murine model of IBD [38], and despite the minor inflammatory mediator phenotype, K8^flox/flox^; Villin-Cre mice also share a notable similarity to IBD including epithelial damage, changes in crypt morphometry, hyperproliferation and declined barrier properties.

We report here that the intestinal epithelial-specific K8-deficient mice are remarkably highly sensitized to AOM-induced colorectal cancer. Based on this data, we can conclude that the tumorigenic phenotype described in the K8^–/–^ is not caused by lack of K8 in the liver where AOM is metabolized [18]. This also supports that the amount of keratins in the colonic epithelia correlates inversely with susceptibility to colitis [30] and inflammation-induced colorectal cancer [39]. A susceptibility for colonic tumorigenesis of keratin-deficient mice is likely consequent to the observed hyperproliferation and proproliferative cell signaling in the colon epithelium described here. The colonocyte K8 loss decreased IL-22BP protein levels and activated STAT3, similar to earlier observations in the K8^–/–^ colon [18]. IL-22BP, also referred to as IL-22RA2, is a soluble high-affinity IL-22 receptor produced by different cell types including epithelial cells in the colon [40, 41] and one of its key roles is to neutralize excessive IL-22 signaling [29]. Here, the activation of the IL-22 pathway after keratin loss is accompanied by a significant increase of IL-22 synthesis on tissue level and slightly elevated circulating concentrations, although significant only in AOM-treated mice. IL-22 is produced by various immune cells [42] and it has several roles, both protective and deleterious, in the colon. IL-22 activates the STAT3 signaling pathway in epithelial cells [43] as was also seen here through upregulation of its target gene. STAT3 is one of the known transcription factors that regulates cell proliferation, tissue regeneration and survival, but it is also involved in the pathogenesis of IBD and CRC [44, 45], thus linking keratin dysfunction with tumorigenesis. Recent studies focusing on the regulation of IL-22 signaling in IBD have concentrated on the role of various immune cell compartments producing IL-22BP [46, 47]. Nevertheless, the close relationship of IL-22BP and intestinal epithelial K8 [18], which is not expressed in immune cells, tempts considering whether the epithelial cell-produced IL-22BP has a more significant role for colon homeostasis than previously assumed. An interesting finding is that IL-18 was downregulated in the intestinal epithelial-specific K8-deficient colon tissue of younger adults, on systemic level in aging mice and reduced on average in K8-deficient AOM-treated mice. The downregulated IL-18 levels may indicate the reduced activity of the inflammasome [48], which may contribute to both the tumor sensitivity and the modest inflammatory responses in K8^flox/flox^; Villin-Cre mice. To this end, we have previously demonstrated that K8 is found in complex with the inflammasome and may, thus, regulate the inflammasome activity [18]. Our result also highlights the role of colon tissue in IL-18 production [49].

Importantly, further linking keratins to colonic proliferation is our data showing that K8 promotes the activity of the cell cycle inhibitor pRb, and when colonocyte K8 is deleted, pRb phosphorylation is increased, promoting cell cycle progression as suggested here by target gene synthesis. How cytoplasmic keratins affect pRb phosphorylation to regulate the cell cycle specifically remains unclear; however it may involve the interactions of colonocyte keratins with the nuclear lamina and lamina-associated proteins [50], which in turn are known to regulate pRb activity [51, 52]. It is also important to note that the keratins can affect proliferation via 14-3-3, a major cell cycle regulator. Indeed, it has been shown that K18 can function as a driver of mitosis by interacting and binding to 14-3-3 [53]. [^18^F]FDG-PET imagining revealed increased metabolic activity in K8^flox/flox^; Villin-Cre mice colons, likely induced by hyperproliferating epithelial cells and low-grade increase in immune cell activity, thus indicating how keratin deficiency modulates metabolism, also suggested previously [16, 54]. Intestinal epithelial K8 deficiency in conditional and full K8-knockout mice led not only to an increase in dividing cells, but also to a shifted cell differentiation program toward goblet cells in a Notch-dependent manner [17]. Interestingly, increases in IL-22 has been shown in intestinal organoids to inhibit Notch signaling, leading to an expanded dividing transit-amplifying cell zone and goblet cell hyperplasia [55], suggesting a colonocyte-intrinsic effect of IL-22 on epithelial proliferation and differentiation and supports a more direct role for K8 in this pathway, while other contributing pathways cannot be excluded. Taken together local keratin deficiency in colon stimulates IL-22 activity and IL-22BP downregulation, thus promoting proliferative and survival signaling, which sensitizes to carcinogenesis. It is noteworthy that major leukocyte involvement is not imperative for this process.

In conclusion, we show that colonocyte keratin filaments have a presumably mechanical, cell-autonomous function in maintaining the intactness of the epithelial barrier and modulating colonic cell cycle and regeneration signaling pathways, maintaining a balanced cell proliferation and consequent renewal of the intestinal epithelium. Balancing the proliferative capacity, colonocyte K8 has a direct or indirect role as a suppressor of tumorigenesis in the colorectum. Future research should also benefit from the here developed fertile and non-lethal K8^flox/flox^; Villin-Cre model for colorectal disease-related pharmaceutical research.

## Materials and methods

### Experimental animals

Transgenic K8^flox/flox^ animals in the C57BL/6 background were generated by Ozgene (Cambridge, MA, USA) (Supplemental Fig. 1) by flanking exon 3 with loxP sites and a neomycin selection segment with FRT sites in C57BL/6 embryonic stem cells. The neomycin segment was then removed using flp recombinase, leaving the loxP sites intact flanking exon 3 (K8^flox/flox^). To then generate a intestinal epithelial-specific K8 knockout mouse, K8^flox/flox^ mice were bred with either Villin-Cre1000 or Villin-CreER^t2^ mice in C57BL/6 background [26]. The conditional mice were maintained by breeding either K8^flox/flox^ mice with K8^flox/−^; Villin-Cre or K8^flox/flox^; Villin-Cre, or by breeding K8^flox/flox^ with K8^flox/flox^; Villin-CreER^t2^. Mice were genotyped by using PuReTaq Ready-To-Go (RTG) PCR Beads (GE Healthcare, UK) and primers 5′-GCGTGGCTTTGGGATTTAGATTAG-3′ and 5′-CCTCCAGCCATGTTTCTTTATCTC-3′ (for the flox transgene) and 5′-GCGATCGCTATTTTCCATGA-3′ and 5′-TCGATGCAACGAGTGATGAG-3′ (for the Cre transgene). Mice were split into two age groups according to their colon phenotype, referred here as adult 3- to 8 month-old mice and old 10- to 14-month-old mice as indicated in figure legends. Age- and sex-matched adult full body K8^−/−^ mice and their wild-type littermates (in FVB/n background) [11] were also studied.

Mice were housed at the Central Animal Laboratory of University of Turku and treated according to animal license (3956/04.10.07/2016 and ESAVI/16359/2019) approved by the State Provincial Office of South Finland. Mice were euthanized by CO_2_ inhalation, followed by intracardiac puncture for blood collection. Colon was excised and the length measured. Tissues were either snap frozen and stored in liquid nitrogen, fixed in 4% PFA, followed by paraffin embedding or embedded in Optimal Cutting Temperature compound (OCT) (Sakura Finetek, Netherlands) and kept at − 80 °C. Proximal colon (PC) and distal colon (DC) were collected for protein and RNA analysis, histology, and immunohistochemistry. Crudely isolated colonic epithelium (as previously described in [16]), ileum and liver were collected for protein analysis.

### Tamoxifen induced K8 deletion in mice

Tamoxifen (Sigma-Aldrich, CA, USA) solution was prepared by dissolving 30 mg tamoxifen in 0.2 ml EtOH and further diluted to 1.8 ml with corn oil (Sigma-Aldrich, CA, USA) to reach a concentration of 15 mg/ml. Vehicle solution with EtOH and corn oil was prepared. Adult 3- to 5-month-old K8^flox/flox^ and K8^flox/flox^; Villin-Cre-ER^t2^ mice received a daily 1.5 mg tamoxifen dose or vehicle solution intraperitoneally for 5 consecutive days. Mice were killed 25 days after the first injection.

### Azoxymethane induced colon carcinogenesis

For the AOM-induced CRC model, 5-month-old K8^flox/flox^ and K8^flox/flox^; Villin-Cre mice were intraperitoneally injected with a 10 mg/kg dose of AOM (Sigma-Aldrich, St. Louis, MO) in 0.9% NaCl solution once per week for 4 weeks and killed 20 weeks after the first AOM administration. The changes in mouse health and body weights were monitored weekly. After killing, colonic tumors were macroscopically counted, identified as spherical and their volumes (*V*) were quantified assuming a globular shape, using the formula *V* = 4/3 × π × *r*^3^, and processed for histology.

### Disease activity analysis

Disease activity was measured as stool consistency and rectal bleeding in K8^flox/flox^; Villin-Cre mice, tamoxifen-induced K8^flox/flox^; Villin-Cre-ER^t2^ mice and in AOM-treated mice similarly to that previously described [30]. Stool consistency was scored as 1 = normal; 2 = formed but soft; 3 = slightly loose; 4 = liquid or unable to excrete. Bleeding was graded as 0 = none; 1 = small amounts of blood in stool; 2 = blood found throughout pellet; 3 = clotted blood at anus; 4 = fresh bleeding.

### FITC–dextran FD4 in vivo permeability assay

Fluorescein isothiocyanate-conjugated dextran (4 kDa; FITC–dextran, FD4, TdB Consultancy AB, Uppsala, Sweden) was used to assess the intestinal permeability by administering 60 mg/kg body weight FD4 by oral gavage to adult mice (*n* = 4 for K8^flox/flox^ and 6 for K8^flox/flox^; Villin-Cre) that had been fasted for 8 h. Mice were killed 6 h after FD4 administration, blood was collected by cardiac puncture, and blood samples were centrifuged (1500 rpm, 10 min) to collect serum. Blood and serum samples were kept in the dark. FD4 values were measured from serum diluted 1:5 in PBS using Victor2 plate reader (PerkinElmer Inc., Finland, Turku) at excitation 493 nm and emission 520 nm. The FD4 concentrations were calculated based on a standard curve.

### Histologic evaluation and immunohistochemistry

Radially cut colon samples, liver, gallbladder, kidney and uterus samples were fixed with 4% PFA, pH 7.4, and embedded in paraffin prior to cutting into 4 µm-thick sections for hematoxylin and eosin (HE) and periodic acid Schiff (PAS) staining (Turku Center for Disease Modeling histology service unit). Mean crypt length was quantitated from ten HE-stained crypts per mouse from both proximal and distal parts of the colon. Cryptless area indicates the parts of colonic mucosa with clear epithelial erosion and the lack of crypt structures presented as a cryptless % of the total colon perimeter, calculated according to mucosae muscularis. The number of goblet cells per colon crypt was counted in 20 randomly selected PAS-stained crypts with sagittal orientation. The goblet cell density was measured by dividing the goblet cell number per crypt with the total top-to-bottom length of each respective crypt.

OCT-embedded samples were cut into 6 μm sections with a Leica CM3050S or CM1950 Research Cryostat (Leica Microsystems, Wetzlar, Germany) and fixed with − 20 °C acetone for 10 min and stained as previously described [56]. Immunofluorescent staining of sections was performed using the antibodies listed in Supplementary Table 1 and counterstained using the nuclear markers DRAQ5 (Cell Signaling, MA, USA) or DAPI (Invitrogen, CA, USA). Ki67 (SolA15) was detected using DAB-conjugated secondary antibody with hematoxylin as counterstaining (Turku Center for Disease Modeling histology service unit). Images were captured using Zeiss LSM780 and Leica TCS SP5 (Wezla, Germany) confocal microscopes (Jena, Germany), and Pannoramic 1000 and Pannoramic MIDI slide scanners (3DHISTECH, Budapest, Hungary). The density of dividing epithelial cells was measured by counting the number of Ki67-positive mitotic bodies in the colon epithelia per mm^2^ of the respective epithelial area using the CaseViewer digital microscopy application (3DHISTECH). MPO-positive cells were calculated using the QuPath v0.2.3 program [57]. Briefly, the area inside (luminal of the mucosae muscularis) was manually selected. Cells were identified according to nuclear staining and size using cell detection tool, and MPO positivity was determined based on cellular intensity of AF568 (2nd antibody for MPO), using a pre-determined cutoff value to exclude possible background (*n* = 6, both sexes equal numbers). The number of lymphoid cell aggregates and their area in the colon were calculated in longitudinally cut HE-stained full colon samples.

### SDS-page and western blot

Total tissues or crudely isolated colonic epithelial tissues were homogenized in 0.187 M Tris–HCl, pH 6.8, 3% SDS, 5 mM EDTA, 1 × complete protease inhibitor cocktail (Roche Switzerland) and 1 mM phenylmethylsulfonyl fluoride on ice. Protein concentrations were afterward measured using Pierce BCA protein assay kit (Thermo Fisher Scientific, Wlatham, MA, USA). Samples were diluted to 5 µg protein/10 µl with 3 × Laemmli sample buffer (30% glycerol, 3% SDS, 0.1875 M Tris–HCl, pH 6.8, 0.015% bromophenol blue and 3% β-mercaptoethanol) and separated on 6–15% SDS–polyacrylamide gels together with iBright™ Prestained Protein Ladder (Thermo Fisher Scientific, Wlatham, MA, USA) or Precision Plus Protein Dual Color Standards (Bio-Rad, CA, USA). Proteins were transferred to polyvinylidene fluoride membranes and analyzed by western blotting (primary and secondary antibodies are listed in Supplementary Tables 3 and 4), and protein bands were quantified using ImageJ software (National Institutes of Health, MD, USA) as previously described [58] and normalized to loading controls.

### Gene expression analysis

RNA was isolated from total colon lysate with a NucleoSpin® RNA kit (Macherey–Nagel, Germany) and the RNA quality was analyzed on  a 1% agarose gel. RNA samples were reverse transcribed into cDNA using cDNA synthesis kit (Promega, Madison, WI). Genes of interest (Supplementary Tables 5 and 6) were amplified using QuantStudio™ 3 real-time PCR system (Applied Biosystems™, CA, USA) with Taqman gene expression assays or designed primers and SensiFAST SYBR Hi-ROX Kit (Meridian Bioscience, Cincinnati, OH, USA). The gene expressions were normalized to both β-actin and ribosomal S18 and the difference between genotypes analyzed according to the delta delta Ct method.

### Cytokine protein measurements

Concentrations of circulating IL-1β, IL-5, IL-6, IL-18 IL-22, IL-25, CCL2, Interferon γ (IFNγ), and TNFα in serum were analyzed with a Procartaplex multiplex assay (ThermoFisher Scientific, Vienna, Austria) according to the manufacturer’s instructions. Serum samples were centrifuged (9600 × *g*, 10 min) prior to analysis to remove debris, and cytokine concentrations were measured using the Luminex 200 system (Luminex Corporation, Austin, TX, USA) and quantified based on protein standards included in the assay kit.

### In vivo positron emission tomography imaging

K8^flox/flox^; Villin-Cre and K8^flox/flox^ control mice (adult 7–8 months old females) with an average body weight of 26.1 and 33.3 g, respectively, were anesthetized with 2.5% isoflurane, and body temperature was maintained using a heating pad. After a transmission scan for attenuation correction using the computed tomographic modality (X-Cube, Molecubes, Gent, Belgium), a positron emission tomography (PET) scan was acquired in three-dimensional list mode (β-Cube; Molecubes, Gent, Belgium). Mice were injected with 8.5 (7.8–9.2) MBq of [^18^F]FDG into a tail vein (Turku PET Centre, Turku, Finland). Static 20-min long scans were acquired and reconstructed with an OSEM two-dimensional iterative algorithm. After the scan, mice were killed, blood samples were collected through cardiac puncture, and tissues were removed and weighed. Samples were measured for ^18^F-radioactivity in a gamma counter (2480 WIZARD2, PerkinElmer, Turku, Finland). The measured radioactivity was corrected for decay and background, and expressed as the standardized uptake value (SUV).

### Statistical analysis

Statistical significance between two groups was determined after unpaired *t* test. *P < 0.05; **P < 0.01; ***P < 0.001; ****P < 0.0001. In a comparison of more than two groups, one-way analysis of variance and Tukey’s post hoc or Kolmogorov–Smirnov test were utilized. Prism and Adobe Illustrator were used to generate graphs.

## Supplementary Information

Below is the link to the electronic supplementary material.

Supplementary file1 (PDF 2458 KB)

Supplementary file2 (PDF 279 KB)

## Data Availability

All data generated or analyzed during this study are included in this published article and its supplementary information files.

## Abbreviations

AOM: Azoxymethane
CCL-2: Chemokine (C–C motif) ligand 2
CRC: Colorectal cancer
DC: Distal colon
FDG: Fluoroglucose
HE: Hematoxylin and eosin
IBD: Inflammatory bowel disease
IF: Intermediate filament
IFNγ: Interferon γ
K: Keratin
MPO: Myeloperoxidase
FLN: Full-length Notch 1
NICD: Notch1 intracellular domain
OCT: Optimal cutting temperature compound
p: Phosphorylated
PAS: Periodic acid Schiff
PC: Proximal colon
PET: Positron emission tomography
pRb: Retinoblastoma protein
SEK: Simple epithelial keratins
SUV: Standardized uptake value
Villin-Cre: Villin-Cre1000
