# Supplementary material for: Targeted deletion of keratin 8 in intestinal epithelial cells disrupts tissue integrity and predisposes to tumorigenesis in the colon
Source: Cell Mol Life Sci. 2021 Dec 24;79(1):10. doi: 10.1007/s00018-021-04081-5 (PMC8709826; doi:10.1007/s00018-021-04081-5)
Supplement: Supplementary file 1 — Supplementary file1 (PDF 2458 KB) [file 18_2021_4081_MOESM1_ESM.pdf]

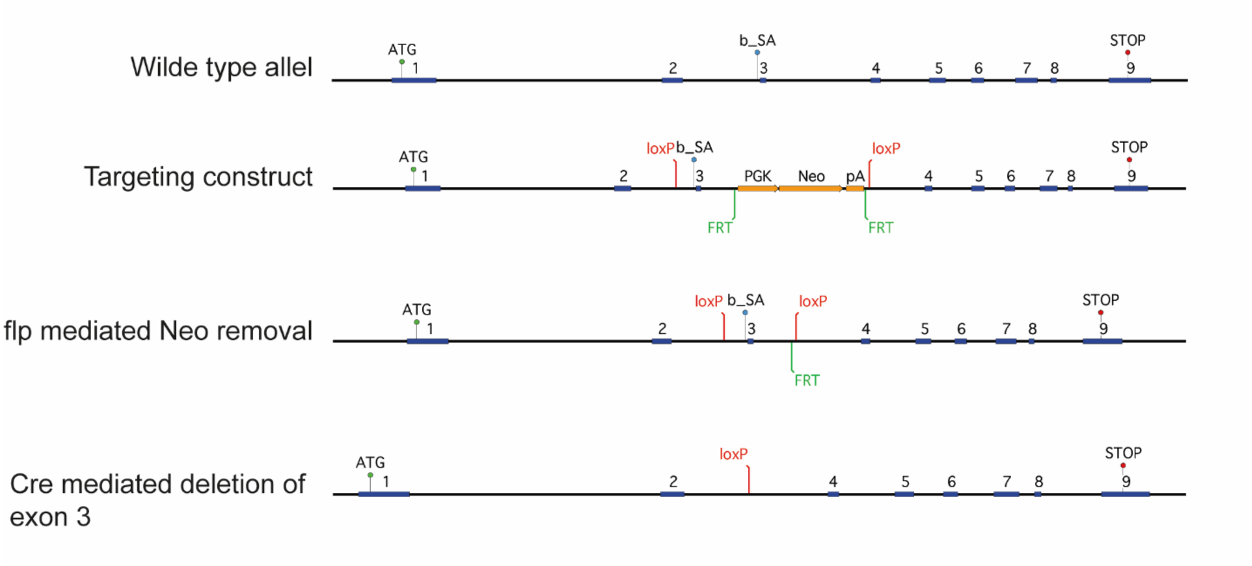

Supplemental Fig. 1 Generation of K8$^{flox/flox}$ mice K8$^{flox/flox}$ mice were generated by targeting exon 3 of the mouse wild type Krt8 allele with loxP sites. The selection marker (Neo) was removed by flp recombinase at recognition sequence sites FRT. Krt8 exon 3 will consequently be deleted when crossing of K8$^{flox/flox}$ mice with Cre-expressing mouse strains.

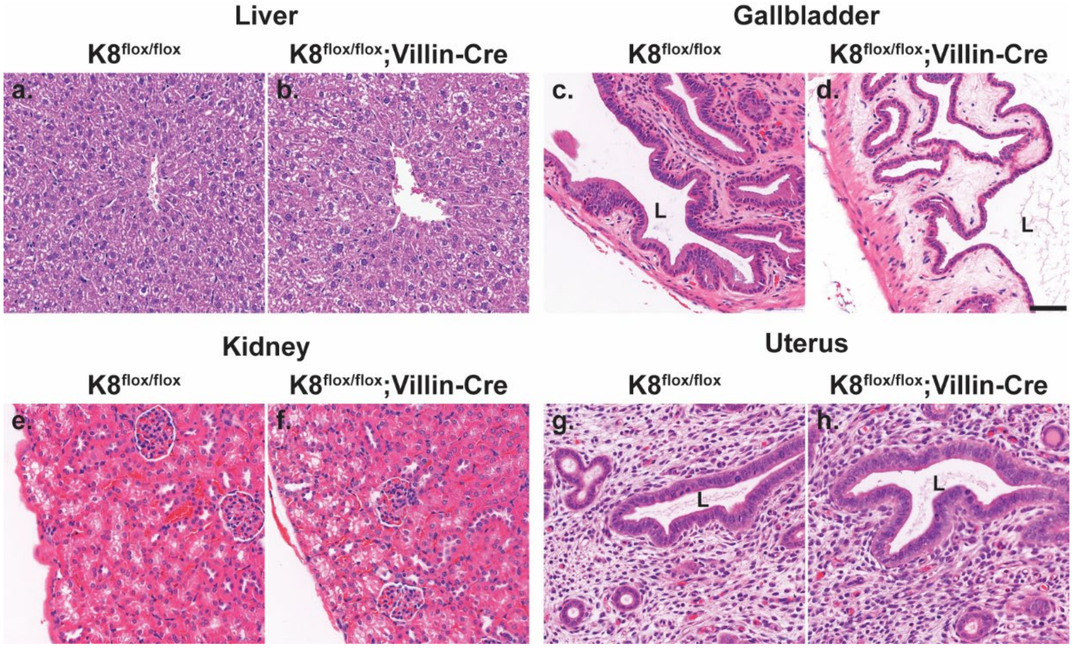

Supplemental Fig. 2 Cre-Villin mediated K8 loss does not affect liver, kidney, uterus or gallbladder epithelial histology Representative HE stainings of liver (a-b), gallbladder (c-d) kidney (e-f) and uterus epithelia (g-h) from $K8^{flox/flox}$ and $K8^{flox/flox}$;Villin-Cre mice. Scale bar 50 µm.

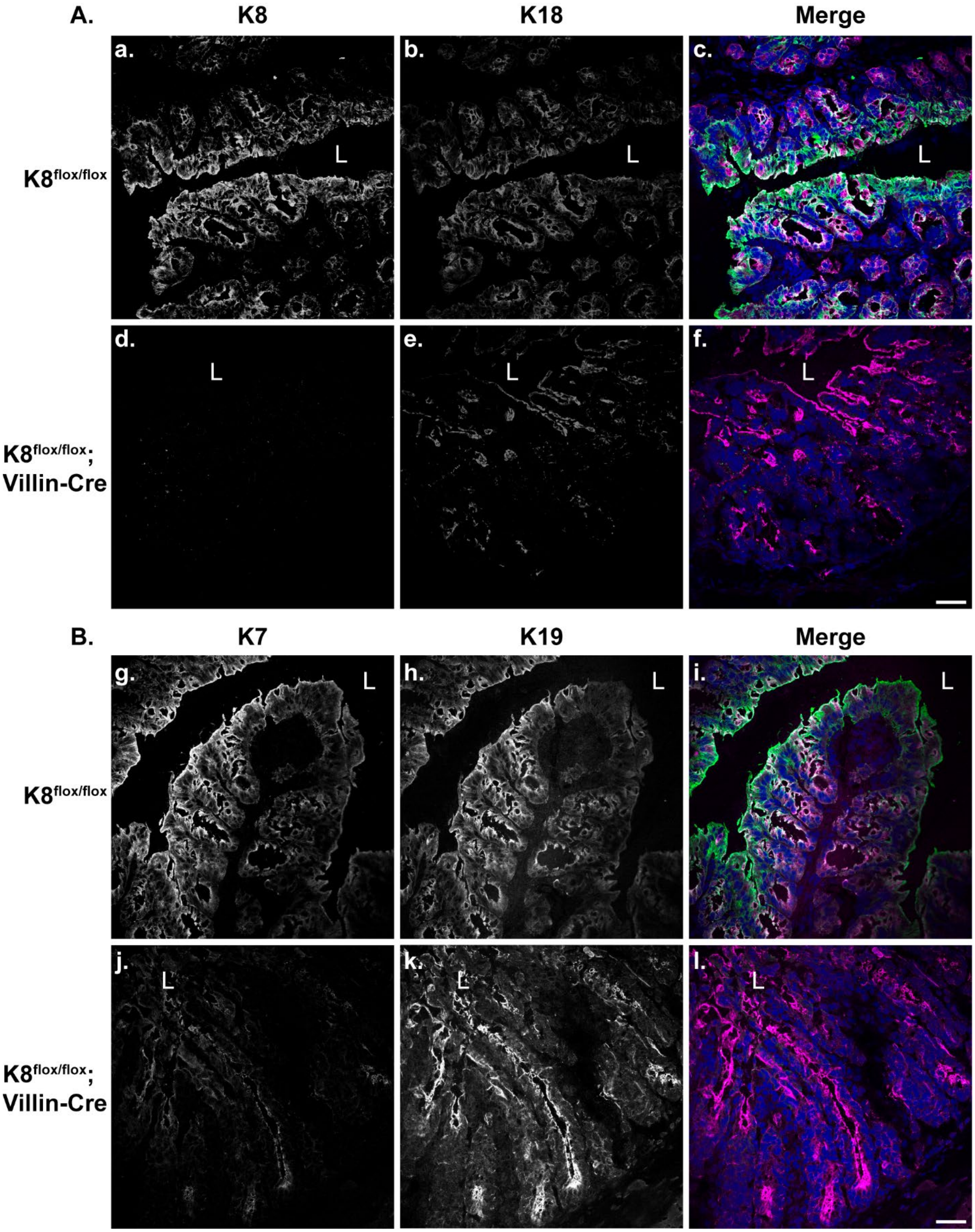
A.
K8
K18
Merge
a.
b.
c.
L
L
L
K8flox/flox
d.
e.
f.
L
L
L
K8flox/flox;
Villin-Cre
B.
K7
K19
Merge
g.
h.
i.
L
L
L
K8flox/flox
j.
k.
l.
L
L
L
K8flox/flox;
Villin-Cre

Supplemental Fig. 3 K8 deficiency in intestinal epithelia decreases and relocalises all colonocyte keratins $K8^{flox/flox}$ and $K8^{flox/flox}$;Cre-Villin mouse colon tissue cryosections were immunostained for A) K8 (green) and K18 (magenta) or B) K7 (green) and K19 (magenta). DNA (blue) was stained with DRAQ5. Merged images shown in c, f, i, l. L = lumen. Scale bar 40 µm. Images are representative of n=6.

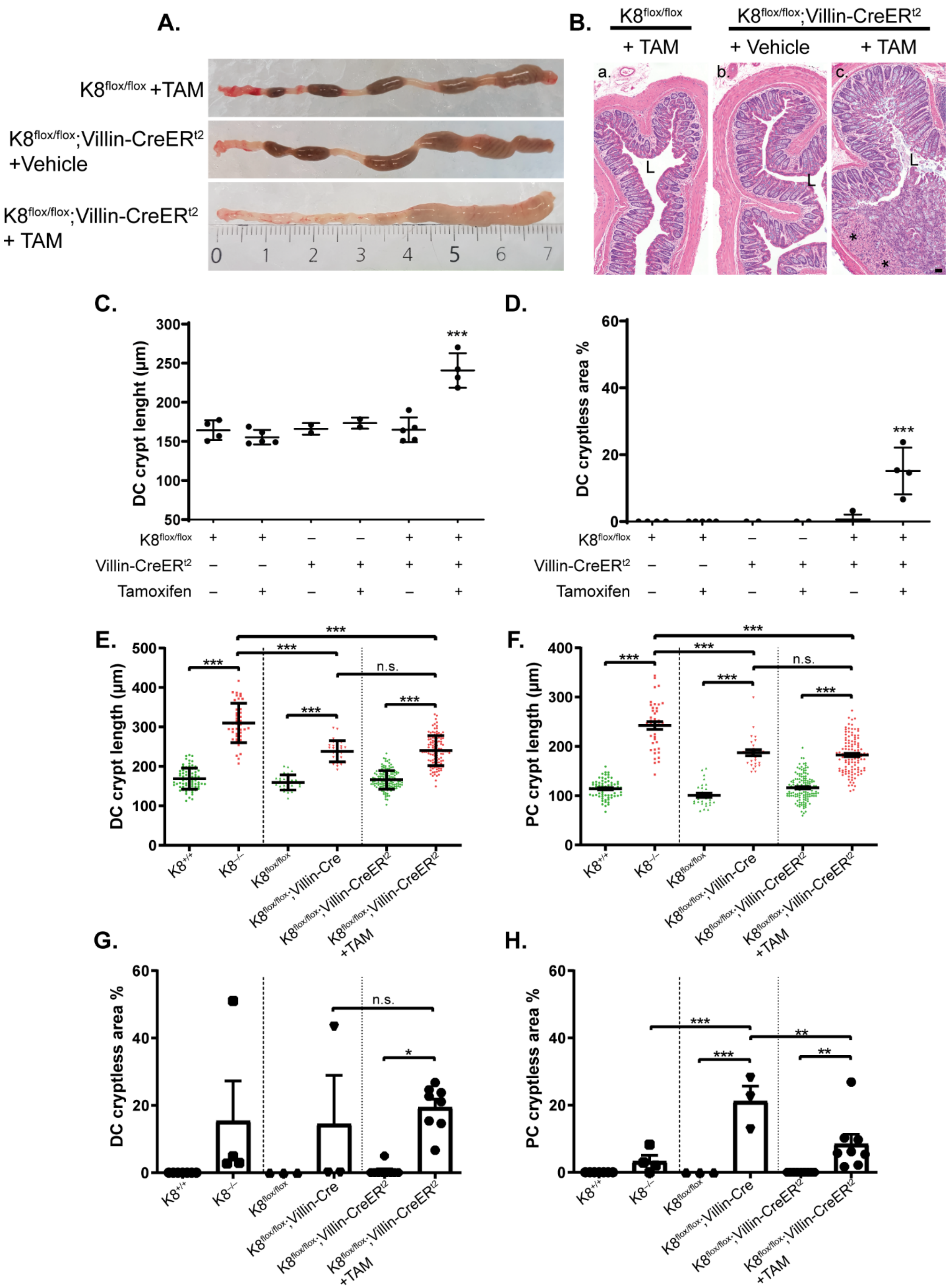

A.
K8flox/flox +TAM
K8flox/flox;Villin-CreERt2 +Vehicle
K8flox/flox;Villin-CreERt2 + TAM
0 1 2 3 4 5 6 7
B.
K8flox/flox
K8flox/flox;Villin-CreERt2
+ TAM
+ Vehicle
+ TAM
a.
b.
c.
L
C.
DC crypt lenght (µm)
***
K8flox/flox
Villin-CreERt2
Tamoxifen
D.
DC cryptless area %
***
E.
DC crypt length (µm)
***
n.s.
F.
PC crypt length (µm)
G.
DC cryptless area %
n.s.
*
H.
PC cryptless area %
**
K8+/+
K8−/−
K8flox/flox
K8flox/flox;Villin-Cre
K8flox/flox;Villin-CreERt2
K8flox/flox;Villin-CreERt2 +TAM

Supplemental Fig. 4 Tamoxifen treated K8$^{flox/flox}$;Villin-CreER^{t2} mice show similar colon disease phenotype to K8$^{flox/flox}$;Villin-Cre mice A) Representative colon images of K8$^{flox/flox}$ and K8$^{flox/flox}$;Villin-CreER^{t2} 25 days after first tamoxifen treatment. B) Representative colon histology images show increased colon crypt length as well as cryptless areas, observed in K8$^{flox/flox}$;Villin-CreER^{t2} mice treated with tamoxifen. * indicates crypt loss. L = lumen. Scale bar = 50 μm C-D) K8$^{flox/flox}$, Villin-CreER^{t2} and K8$^{flox/flox}$;Villin-CreER^{t2} mice treated with or without tamoxifen (25 days after first injection) were analyzed for colon crypt length (C) and cryptless areas (D). Dots indicate individual mice, and data is given as mean +/- SD. Tamoxifen or vehicle treatment as well as mouse genotype are indicated with + and -. P-values represent difference between genotypes and were determined after one-way ANOVA followed by post hoc Tukey multiple comparison test, with significances shown as *** = $P < 0.001$. n=3-5. E-H) Comparisons of colon crypt length and cryptless areas between K8$^{-/-}$ and both conditional K8 knockout mouse models. Dots indicate individual crypts in E-F, n=28-140, and individual mice in G-H, n=3-8, between the ages 4-6 months, and data is presented as mean +/- SD. P-values represent difference between genotypes and were determined after one-way ANOVA followed by post hoc Tukey multiple comparison test, with significances shown as n.s. = not significant, * = $P < 0.05$, ** = $P < 0.01$, *** = $P < 0.001$.

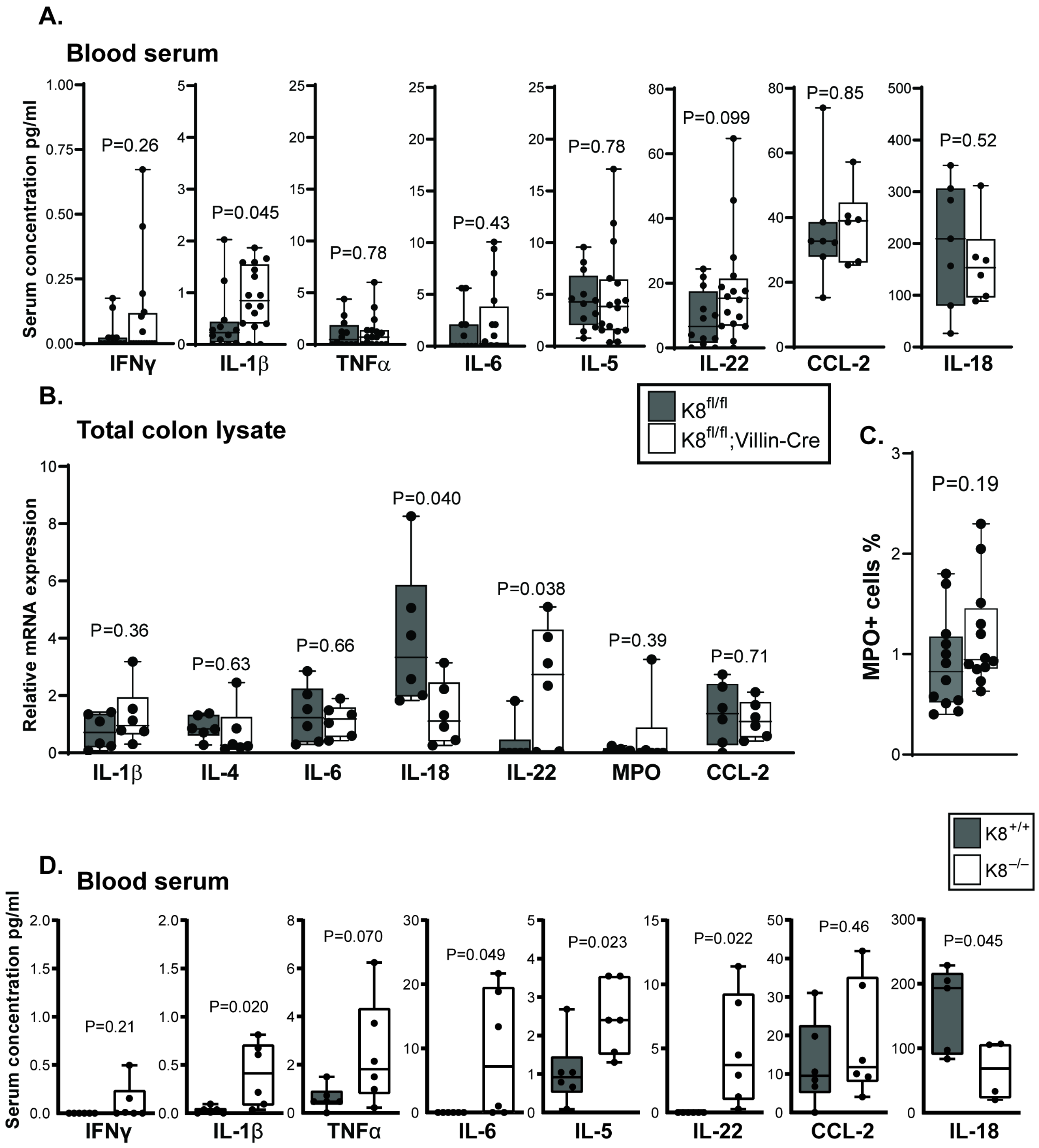

Supplemental Fig. 5 Conditional K8 loss does not provoke a significant inflammatory response but modulates IL-18 and IL-22 synthesis A) Concentrations of circulating IFNχ, IL-1β, TNFα, IL-6, IL-5, IL-22, CCL-2 and IL-18 in serum of K8$^{flox/flox}$and K8$^{flox/flox}$;Villin-Cre mice. B) The fold changes in colon tissue lysate gene expression of *Il1b*, *Il4*, *Il6, Il18, Il22, Mpo* and *Ccl2* in K8$^{flox/flox}$, and K8$^{flox/flox}$;Villin-Cre

mice. C) % of MPO positive cell inside colon muscularis mucosae, classified digitally using the QuPath program "positive cell detection" tool. n=6 mice per group, and two tissue samples from each mouse (one from both distal and proximal colon parts were analyzed). D) The concentrations of circulating IFNχ, IL-1β, TNFα, IL-6, IL-5, IL-22, CCL-2 and IL-18 in serum of $K8^{+/+}$ and $K8^{-/-}$ mice. In this figure boxes extend from 25th to 75th percentiles and line represents median expression value and whiskers represent min and max values and individual mice values are represented as dots. P-values represent difference between genotypes and were determined using student's T test.

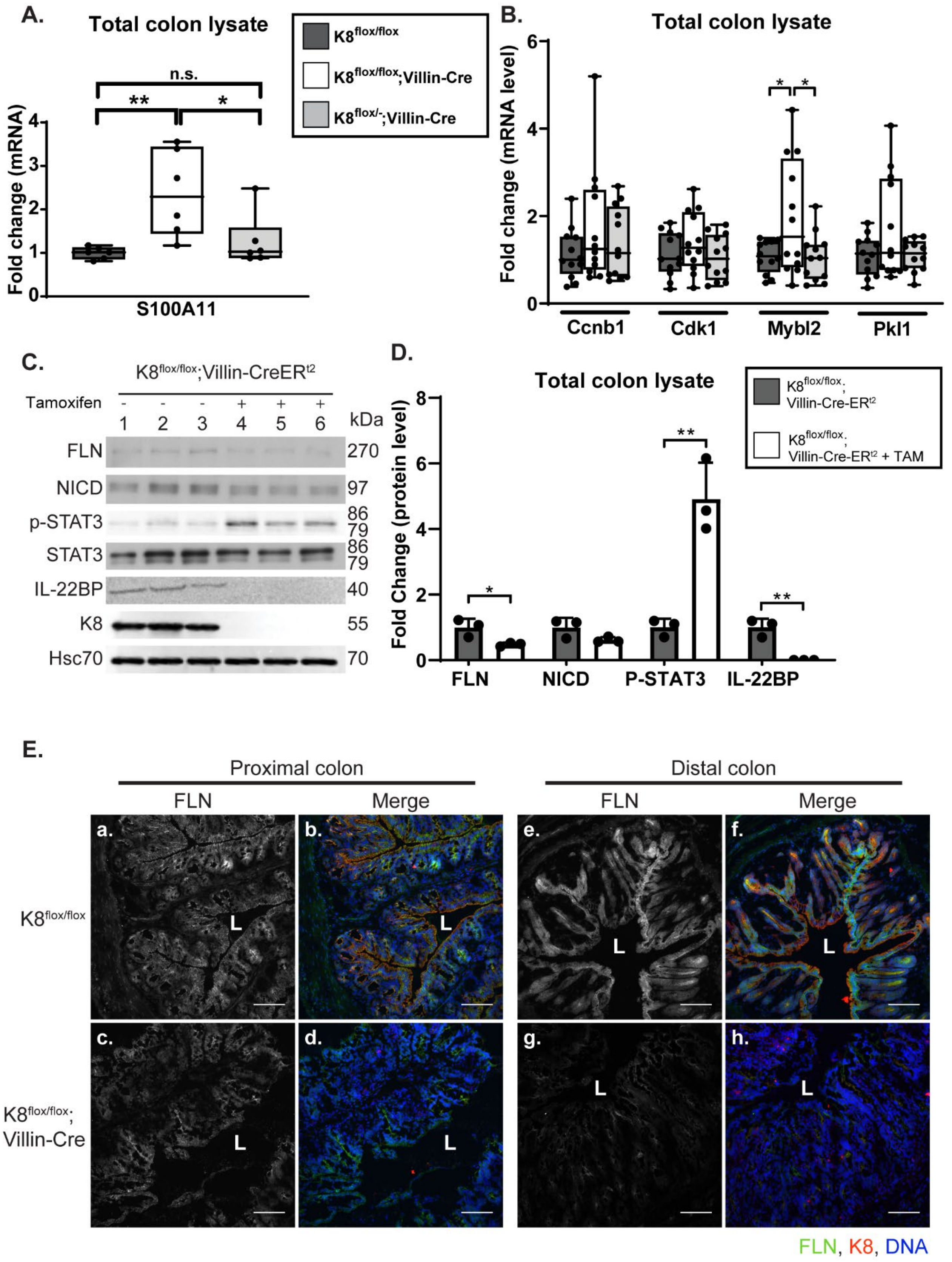

A.
Total colon lysate
n.s.
**
*
Fold change (mRNA)
S100A11
K8flox/flox
K8flox/flox;Villin-Cre
K8flox/-;Villin-Cre
B.
Total colon lysate
Fold change (mRNA level)
Ccnb1
Cdk1
Mybl2
Pkl1
C.
K8flox/flox;Villin-CreERt2
Tamoxifen
kDa
FLN
NICD
p-STAT3
STAT3
IL-22BP
K8
Hsc70
D.
Total colon lysate
Fold Change (protein level)
K8flox/flox; Villin-Cre-ERt2
K8flox/flox; Villin-Cre-ERt2 + TAM
FLN
NICD
P-STAT3
IL-22BP
E.
Proximal colon
Distal colon
FLN
Merge
K8flox/flox
K8flox/flox; Villin-Cre
FLN, K8, DNA

Supplemental Fig. 6 Tamoxifen treated K8$^{flox/flox}$;Villin-CreER^{t2} have increased p-STAT3 and near complete loss of IL-22BP and K8$^{flox/flox}$;Villin-Cre mice display increased STAT3 target gene expression A-B) mRNA analyzed from total colon samples by qRT-PCR show increased STAT3 target gene S100A11 levels in K8$^{flox/flox}$;Villin-Cre mice colon (A) and pRb target genes Ccnb2, Cdk1, Mybl2 and Pkl1 (B) in which boxes extend from 25th to 75th percentiles and line represents median expression value and whiskers represent min and max values with individual mice values shown as dots. C) Total colon lysates from vehicle K8$^{flox/flox}$;Villin-CreER^{t2} (lane 1-3) and tamoxifen treated (+, 25 days after first injection) K8$^{flox/flox}$;Villin-CreER^{t2} (lane 4-6) mice (n=3) were immunoblotted and signal quantified (D) for FLN, NICD, p-STAT3, STAT3, IL-22BP and K8. Hsc70 was used as a loading control. The graph represent mean ± SD with individual mice values shown as dots. The significance was determined after one-way ANOVA followed by post hoc Tukey multiple comparison test, except in D) by student's T test. n.s. = not significant, $P<0.05$ and ** = $P < 0.01$. E) K8$^{flox/flox}$ and K8$^{flox/flox}$;Villin-Cre mouse proximal and distal colon tissue cryosections were immunostained for FLN (green) and K8 (red). DNA (blue) was stained with DRAQ5. Merged images shown in b, d, f, h. L = lumen. Scale bar 100 µm. Images are representative of n=3 mice.
