# Supplementary material for: Targeted deletion of keratin 8 in intestinal epithelial cells disrupts tissue integrity and predisposes to tumorigenesis in the colon
Source: Cell Mol Life Sci. 2021 Dec 24;79(1):10. doi: 10.1007/s00018-021-04081-5 (PMC8709826; doi:10.1007/s00018-021-04081-5)
Supplement: Supplementary file 2 — Supplementary file2 (PDF 279 KB) [file 18_2021_4081_MOESM2_ESM.pdf]

Supplementary Table 1. Comparison of disease phenotype between full body and intestinal epithelial-specific K8 knockout mouse models.

	Keratin 8 knockout mouse models			
	Full K8 knockouts (FVB/n background)*	Intestinal epithelial cell K8 knockouts (C57BL/6 background)**		
**Disease phenotype**	$K8^{-/-}$	$K8^{flox/flox}$;Villin-$CreER^{t2}$***	$K8^{flox/flox}$;Villin-Cre	**References**
**Colon inflammation**	Yes, early onset	n.d.	Yes, in aging mice	Habtezion et al., 2005; Toivola et al., 2004 Asghar et al., 2014
**Colonic epithelial hyperproliferation and increased crypt length**	Yes	Yes	Yes	Baribault et al. 1994; Toivola et al., 2004
**Cryptless/damaged areas**	Yes	Yes	Yes	Asghar et al., 2015
**Intestinal barrier compromised**	Yes	n.d.	Yes	Misiorek et al., 2016
**Diarrhea**	Yes	Yes	Yes	Toivola et al., 2004
**Enhanced colorectal cancer susceptibility**	Yes	n.d.	Yes	Misiorek et al., 2016
**Rectal prolapse**	Observed	none	Observed	Baribault et al. 1994
**Defects in other epithelial organs**	Yes	n.d.	none	Tao et al., 2003; Jaquemar et al., 2003; Mathew et al., 2013; Alam et al., 2013; Odaka et al. 2013; Silvander et al. 2017
**Embryo lethality**	50 %	none	none	Baribault et al. 1994
**Female infetitility**	Yes	none	none	Baribault et al. 1994

*C57Bl/6, $K8^{-/-}$ mice are 95% embryo lethal with 100% female infertility (Baribault et al. 1993); **results from this study; ***25 days after tamoxifen treatment; n.d. not determined

Supplementary Table 2. Summary of K8 dependet cellular a+C4:G19nd molecular phenotypes in the colonic epithelium and serum of full body and the intestinal epithelium specific K8 knockout mouse				
Molecular phenotype		Keratin 8 knockout mouse models		References
		Full K8 knockouts (FVB/n background)	Intestinal epithelial cell K8 knockouts (C57BL/6 background)	
		$K8^{-/-}$	$K8^{flox/flox}$;Villin-Cre	
Simple epithelial keratins	K7	↓	↓*	Asghar et al., 2015; Figure 1
	K18	↓	↓*	
	K19	↓	↓*	
	K20	↓	↓*	
Cell proliferation and tissue repair	IL-22	↑	↑	Misiorek et al., 2016; Figure 5; Supplemental Figure 6
	IL-22BP	↓	↓*	
	p-STAT3	↑	↑*	
	p-pRb	↑	↑	Unpublished; Figure 5
Cell differentiation/ fate	Goblet cells	↑	↑	Lähdeniemi et al., 2017; Figure 5; Supplemental Figure 6
	Villin	↓	↓	
	Notch1	↓	↓*	
Energy metabolism	HMGSC2	↓	↓	Helenius et al., 2015; Unpublished
Serum cytokines	IL-1β	↑	↑	Supplemental Figure 5
	IL-5	↑	n.s	
	IL-6	↑	n.s	
	IL-18	↓	↓**	
	IL-22	↑	n.s	
	IFNγ	n.s	n.s	
	CCL-2	n.s	n.s	
	TNFα	n.s	n.s	

↑ indicates statistically significant increase and ↓ statistically significant decrease compared to respective K8 wild-type control mice; n.s. means non significant change, *$K8^{flox/flox}$;Villin-$CreER^{t2}$ show similar results, ** in aging mice.

Supplementary Table 3. List of primary and secondary antibodies used for western-blot analyses and immunofluorescence

Antibody name	Dilution	Company
Rabbit anti-K7	1:1000/1:100*	Abcam, Cambridge, UK (181598)
Rat anti-K8	1:1000/1:1000*	Developmental Studies Hybridoma Bank, IA, USA (troma I)
Rabbit anti-K18	1:1000/1:1000*	Kind gift from John Eriksson lab
Rat anti-K19	1:1000/1:1000*	EMD Millipore Corporation, CA, USA (MABT913)
Rabbit anti-K20	1:1000	Abcam, Cambridge, UK (97511)
Rabbit anti-Mu2	1:1000	Abcam, Cambridge, UK (272692)
Rabbit anti-Villin	1:1000	Invitrogen, Thermo Fisher Scientific, CA, USA (PA517290)
Rabbit anti-pRb	1:1000	Abcam, Cambridge, UK (181616)
Rabbit anti-pospho-pRb (ser807/811)	1:1000	Cell Signaling, MA, USA (8516)
Rabbit anti-full length Notch 1	1:1000	Cell Signaling, MA, USA (D1E11)
rabbit anti-cleaved Notch 1 (Val1744)	1:1000	Cell Signaling, MA, USA (D3B8)
Rabbit anti-full length Notch 1 (C-20)	1:1000	Santa Cruz, TX, USA (sc-6014)
Mouse anti-STAT3	1:1000	Cell Signaling, MA, USA (9139)
Rabbit anti-phospho-STAT3 (tyr705)	1:1000	Cell Signaling, MA, USA (9145)
Sheep anti-IL-22BP	1:1000	R&D systems (AF2376)
Rat anti-Hsc70	1:1000	Stressgen Bioreagents, MI, USA (SPA-815B)

*dilution factor applied for immunostainings

Supplementary Table 4. List of secondary antibodies used for western-blot analyses and immunofluorescence

Antibody name	Dilution	Company
Anti-rabbit Alexa Fluor 488/680**/800**	1:10000/1:200*	Invitrogen, CA, USA (A21206/A32734/A32735)
Anti-rat Alexa Fluor 488/568/680**	1:10000/1:200*	Invitrogen, CA, USA (A21208/A11077/A21096)
Anti-rabbit IgG-HRP	1:10000	Promega, WI, USA (W401B)
Anti-rat IgG-HRP	1:10000	Cell signaling, MA, USA (7077S)
Anti-mouse IgG-HRP	1:10000	GE Healthcare, UK (NA931V)
Anti-sheep IgG-HRP	1:10000	Sigma-Aldrich, MO, USA (12-342)

*dilution factor applied for immunostainings

**secondary only used for western-blot analyses

Supplemental Table 5. Primer sequences used for TaqMan and SYBR Green used for qRT-PCR gene expression analysis

Gene	Primer sequence	
	Forward primer	Reverse primer
**18S rRNA**	5'-GCA ATT ATT CCC CAT GAA CG -3'	5'-GGG ACT TAA TCA ACG CAA GC -3'
Actb	5'-TGG CTC CTA GCA CCA TGA AGA-3'	5'-GTG GAC AGT GAG GCC AGG AT-3'
Krt7	5'-GGA GAT GGC CAA CCA CAG-3'	5'-GGC CTG GAG TGT CTC AAA CTT-3'
Krt8	5'-TGA ATT TGT CCT CAT CAA GAA GG-3'	5'-GGA TCT CCT CTT CAT GGA TCT G-3'
Krt18	5'-AGA TGA CAC CAA CAT CAT CAC AAG G-3'	5'-CTT CCA GAC CTT GGA CTT CCT-3'
Krt19	5'-TGA CCT GGA GAT GCA GAT TG-3'	5'-CCT CAG GGC AGT AAT TTC CTC-3'
Krt20	5'-AGC TGA GAC GCA CCT ACC AG-3'	5'-TGC GCT CCA GAG ACT CTT TC-3'
Krt23	5'-TCA TGA AGA AAC GCC ATG AG-3'	5'-CCT TGA AGT CAC TCG GCA AG-3'
S100a11	5’-GGG AAG GAT GGA AAC AAC ACT-3’	5’-CAC CAG GAT CCT TCT GGT TC-3’
Plk1	5’-GCA GCA GGA AAC CTC TCA A-3’	5’-GCT GTA GCA AGT CAC TAA GGT-3’
Mybl2	5’-GCT GAT GTC CTC TAC CAT GC-3’	5’-CTG TTA CCC TCT TTG CTA CCT G-3’
Cdk1	5’-GAC TAC AAG AAC ACC TTT CCC A-3’	5’-CGT TTG GCA GGA TCA TAG ACT-3’
Ccnb1	5’-GCC TAT ATT GCA TTT CCT AGT GTG-3’	5’GAA TGA AGC CAA GCA AGA CAT-3’

Supplemental Table 6. TaqMan Gene Expression Assays used for qRT-PCR gene expression analysis

Gene	Expression Assay#
Bact	Mm02619580_g1
Ccl2	Mm00441242_m1
Mpo	Mm01298424_m1
Il18	Mm00434226_m1
Il22	Mm01226722_g1
Il4	Mm00445259_m1
Il6	Mm00446190_m1
Il1b	Mm00434228_m1
